# Underdiagnosis of Hyperparathyroidism in Patients With Nephrolithiasis in a Community Setting

**DOI:** 10.7759/cureus.49290

**Published:** 2023-11-23

**Authors:** Cameron D Moore, Bassil Azam, Helena Do, Kayla Williamson, Amber H Chambers, Muhammad Owais Abdul A Ghani

**Affiliations:** 1 General Surgery, University of Kentucky Bowling Green Campus, The Medical Center, Bowling Green, USA; 2 Trauma and Orthopaedics, Maidstone and Tunbridge Wells NHS Trust, Kent, GBR; 3 Surgery, University of Kentucky Bowling Green Campus, The Medical Center, Bowling Green, USA; 4 General Surgery, Graves Gilbert Clinic, Bowling Green, USA

**Keywords:** hypercalcemia, burden of disease, parathyroid hormone, nephrolithiasis, hyperparathyroidism

## Abstract

Objective

Untreated primary hyperparathyroidism (PHPT) has wide-ranging multisystemic effects. Recent studies based in the US have shown a less than 25% screening rate for PHPT. Our study aims to detect whether similar deficiencies exist in our community healthcare system while quantifying the prevalence of PHPT underdiagnosis and inadequate surgical referrals.

Study design

This retrospective quantitative study enrolled patients aged ≥18 years with imaged-confirmed nephrolithiasis at our healthcare facilities from 2017 to the present (n=2021). Patients with documented calcium levels and kidney/ureter stones were included. Descriptive and univariate analyses were performed.

Results

A total of 2021 subjects met the criteria to be enrolled in the study. 26.6% (n=537) of patients with nephrolithiasis had elevated calcium levels on record. 13.6% (n=73) of hypercalcemic patients were screened for PHPT with an intact parathyroid hormone (PTH). A majority (63%, n=46) of patients with intact PTH had PHPT defined as PTH levels >55 pg/mL. Ultimately, only 19.6% (n=9) of patients with PHPT were referred for surgical intervention, and there was no significant difference in referral rate between patients with PHPT and those without (p=0.913).

Conclusions

PHPT is underdiagnosed in our community, leading to a significantly low rate of surgical referral and delay in management. Implementation of hospital protocols to aid in improving diagnosis and interventions could improve outcomes for PHPT patients.

## Introduction

Untreated primary hyperparathyroidism (PHPT) has a wide range of multisystemic effects, including those related to hypercalcemia and effects on key target organs. Hypercalcemia can lead to polyuria, constipation, anorexia, arrhythmias, dehydration, and ultimately altered mental status. Renal involvement in PHPT leads to hypercalciuria, nephrolithiasis, and possibly reduced renal function [1-4]. Nephrolithiasis and/or nephrocalcinosis is present in up to 21 to 55% of patients with asymptomatic PHPT [5,6]. Skeletal involvement can lead to disorders such as osteitis fibrosa cystica, bone cysts, and brown tumors of the long bones [7,8].

Kidney stones are prevalent in the general population, affecting approximately 1 in 11 people in the United States, with up to 50% of these individuals developing recurrent stones within the next 10 years [9-12]. The etiology of nephrolithiasis is multifactorial, but hypercalciuria is a primary risk factor for stones [13]. Since renal calcium excretion is increased in PHPT, hypercalciuria is generally considered a contributor to the pathophysiology of nephrolithiasis, although the precise relationship between PHPT and nephrolithiasis is not fully understood [14]. Approximately 3-5% of patients developing kidney stones have PHPT, and they face an even higher (~40-fold) risk of recurrent urolithiasis compared to age-matched controls [15].

The treatment of choice for symptomatic PHPT is surgical correction through parathyroidectomy. Removing autonomous parathyroid tissue decreases urine calcium excretion and substantially reduces stone events and recurrence rates, with risk returning to levels comparable to the control population 10 years after surgery [16,17].

Due to the strong, well-documented association between nephrolithiasis and PHPT, guidelines from the American Urological Association and the European Association of Urology recommend measuring serum calcium concentration, followed by serum parathyroid hormone levels, if there is suspicion of PHPT [18,19]. This approach increases the chances of detecting symptomatic PHPT disease, leading to appropriate referrals for surgical correction. However, a recent study has demonstrated that less than 25% of patients with a diagnosis of renal stones and concurrent hypercalcemia are screened for PHPT [19]. With this low screening rate, individuals with symptomatic PHPT are at increased risk of recurrent stones and bone involvement [3].

The disparity in screening for PHPT in patients with nephrolithiasis puts individuals at risk, considering the multisystemic impact of undetected PHPT. Our study aims to investigate whether similar deficiencies exist in our healthcare system by quantifying the prevalence of PHPT underdiagnosis and the subsequent reduction in surgical referrals. If disparities are detected, we plan to propose changes in hospital protocols to assist in the early detection and management of PHPT, thereby improving outcomes for these patients.

## Materials and methods

This study is a retrospective review sourced from the electronic medical record (EMR) systems of our healthcare facilities, including three different hospitals: Medical Center Bowling Green, Franklin, and Scottsville. We included all patients over 18 years of age who presented with nephrolithiasis confirmed on imaging, along with documented calcium levels from 2017 to 2021. The study aims to assess the underdiagnosis of hyperparathyroidism in patients with nephrolithiasis in a community setting. Data collected included demographics (age and gender), laboratory results (serum calcium levels), imaging results (presence of kidney/ureter stones), and clinical history (symptoms of hyperparathyroidism). We also gathered data on intact parathyroid hormone levels and surgery referral rates (endocrine and general surgery).

Descriptive statistics were utilized to summarize the demographic characteristics of the study population. Univariate analysis examined the rates of hyperparathyroidism workup and surgical referral rates. Categorical variables were analyzed using the Chi-square test, while non-parametric tests (Mann-Whitney U) were used for continuous data given their non-normal distribution. Statistical analysis was performed using IBM SPSS Statistics for Windows, Version 26 (Released 2019; IBM Corp., Armonk, New York).

The study was approved by the Institutional Review Board of Med Center Health, The Medical Center at Bowling Green, with the tracking number 21-08-26-Ghani-PHTH. Patient data were kept confidential and anonymous, with no identifying information collected or used in the analysis.

## Results

A total of 2,021 subjects met the criteria to be enrolled in the study. Of these, 537 patients (26.6%) had elevated calcium levels on record, while 1,484 were normocalcemic. Among the hypercalcemic group, 296 (50.5%) were male and 241 (49.5%) were female. In the normocalcemic group, 750 (55.1%) were male and 734 (44.9%) were female. There was no significant difference in sex distribution between hypercalcemic and normocalcemic patients (p=0.69); however, hypercalcemic patients were likely to be older (59 vs. 55; p=0.01; Table 1). Among the hypercalcemic patients, 73 (13.6%) were screened for hyperparathyroidism (PHPT) with an intact parathyroid hormone (PTH) test, while 464 (85.4%) were not screened (Table 1). Of these, 46 patients (63%) were found to have PHPT, defined as PTH levels greater than 55 pg/mL, while 27 patients (37%) had PTH levels within the normal range. There was no significant difference in age between patients who were screened and those who were not.

**Table 1:** Demographics ^a^Column percentages. ^b^Median and interquartile range. *Statistically significant.

	Hypercalcemia (n=537)	Normocalcemia (n=1484)	P-value
Sex: male/female^a^	296 (50.5)/241 (49.5)	750 (55.1)/ 734 (44.9)	0.69
Age^b^	59 (47.5-70.5)	55 (42.5-67.5)	0.01^*^
	Screen PTH	No screen PTH	
Sex: male/female	44 (60.3)/29 (39.7)	252 (54.3)/212 (45.7)	0.34
Age	60 (51.5-68.5)	60 (48-72)	0.49
	PHPT	No PHPT	
Surgical referral	9 (20)	27 (18.5)	0.73

**Figure 1:**
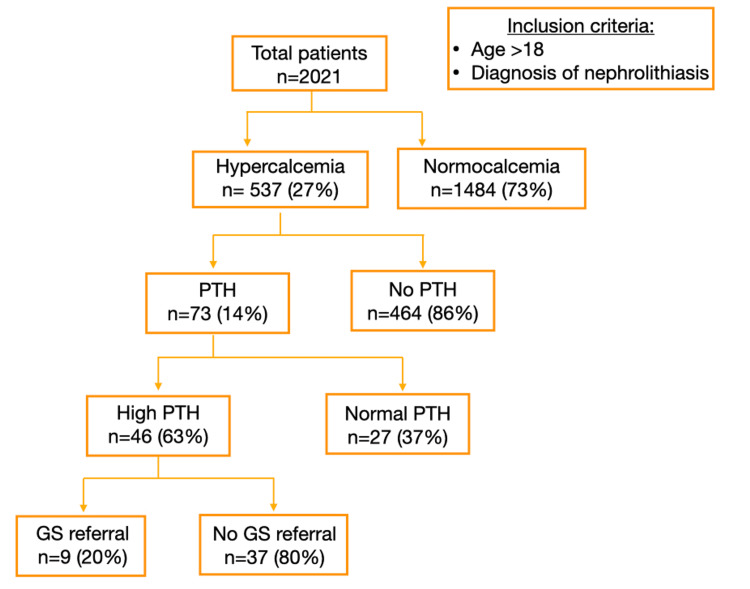
Patient selection and distribution. PTH: parathyroid hormone; GS: general surgery.

Among the 46 patients with PHPT, only 9 (19.6%) were referred for surgical intervention, with the majority of patients (80.4%) not being referred. There was no significant difference in the referral rate between patients with PHPT and those without (p=0.913; Figure 1).

Among the 46 patients with PHPT, only 9 (19.6%) were referred for surgical intervention. There was no significant difference in the referral rate between patients with PHPT and those without (p=0.913; Figure 1).

## Discussion

Our findings align with current literature suggesting that PHPT is not only underdiagnosed but also undertreated in terms of curative surgery, regardless of whether the presentation is symptomatic or asymptomatic [20,21]. Before establishing and implementing protocols to address this issue, it is crucial to understand why this problem exists. Firstly, PHPT can present with subtle, non-classical symptoms such as neuropsychiatric and cognitive disorders, musculoskeletal pain, or gastrointestinal tract dysfunction, requiring clinicians to maintain a high index of suspicion in the evaluation of this disease [22]. The low referral rate for surgical intervention among patients with PHPT suggests that there may be a lack of awareness and understanding of the importance of early detection and treatment of this condition, not only among Emergency Department providers but also in primary care settings. There may be a misperception that PHPT has a low impact on overall health outcomes, leading to its management being deprioritized [21].

Another aspect of this issue is that patients with early or mild disease are not being offered surgical intervention or are being lost in follow-up. Symptom-free patients who do not meet the criteria for parathyroidectomy, or who choose not to undergo surgery, should be reassessed annually for symptoms, hypercalcemia, and osteoporosis. Patients who are unable or unwilling to undergo surveillance should be offered parathyroidectomy, as mild disease may progress [23]. Medical therapy for PHPT, such as the use of cinacalcet, a calcimimetic, is less efficacious and cost-effective than parathyroidectomy and should be limited to patients who are not operative candidates [24].

A study from Denmark questioned the reliance on biochemical parameters (plasma phosphate, calcium, PTH, alkaline phosphatase, creatinine, calcitriol) as predictors of renal calcifications in patients with PHPT, indicating that routine screening with a CT scan is necessary to identify and ensure proper surgical referral [25]. This is particularly crucial for patients who may be experiencing “silent” (asymptomatic) kidney stones, as this subset of patients may have a more severe form of the disease [26]. Furthermore, a separate study from a large tertiary center in Denmark demonstrated that preoperative stone events increase the risk of postoperative stones, further advocating for early identification and management [17]. Additionally, a study from Australia found the Pasieka Illness Questionnaire (PIQ), a disease-specific outcome tool that accounts for non-classical symptoms, to be very effective in both screening and proving the effectiveness of surgical intervention [27]. This tool could theoretically be easily utilized in emergency and primary care settings.

The limitations of this study include its retrospective design and the fact that it was conducted at a single healthcare facility, which may limit the generalizability of the findings. Nevertheless, this study provides important insights into the underdiagnosis and undertreatment of PHPT in patients with nephrolithiasis in a community setting. Further research is needed to better understand the factors influencing the decision to refer for surgical intervention in patients with PHPT and to develop strategies to improve the diagnosis and management of this condition in this patient population.

## Conclusions

Strategies to improve the identification and referral of patients with PHPT for surgical intervention are needed to ensure that these patients receive appropriate and timely care. Ultimately, improving the diagnosis and management of PHPT in this patient population has the potential to improve patient outcomes and reduce the burden of disease in the community. There is also significant cost-saving potential in lowering the hospital visits associated with recurrent nephrolithiasis. To address these gaps in care, we plan to implement emergency room order sets that include parathyroid hormone testing for patients with kidney stones and to establish a reflex lab protocol for triggering a comprehensive workup for PHPT. These interventions have the potential to improve the identification and management of PHPT in patients with nephrolithiasis and to ensure that these patients receive appropriate and timely care.
